# Draft genome sequence of an *Aliiglaciecola* species isolated from the macroalga *Ulva*

**DOI:** 10.1128/mra.01288-24

**Published:** 2025-08-26

**Authors:** Olivia C. Macrae, Chelsea J. Vickers

**Affiliations:** 1 Center for Biodiscovery, School of Biological Sciences, Victoria University of Wellington 8491, Wellington, New Zealand; 2 School of Science, University of Waikato 3717, Hamilton, New Zealand; California State University San Marcos, San Marcos, California, USA

**Keywords:** algae, symbiont, marine microbiology

## Abstract

We report the 4.4 Mb genome sequence of a species of *Aliiglaciecola*, designated *Aliiglaciecola* sp. nov. SL4 (for “sea lettuce 4”). This bacterium was isolated and sequenced during studies of marine macroalgal relationships. Signatures of complex polysaccharide degradation capacity encoded in this genome represent potentially novel microbial metabolic capacity.

## ANNOUNCEMENT

Sea lettuce (*Ulva* sp.) is ubiquitous along the world’s coastlines and is the subject of extensive research for high-value bioactive derivatives (1–5). To explore the symbiotic relationships of this high-value algae, we isolated an epiphytic bacterium associated with *Ulva*. This bacterium demonstrates capacity for ulvan utilization, reported by growth in the presence of ulvan as the sole carbon source. This prompted bacterial genome sequencing to elucidate genomic components contributing to ulvan utilization. Genome sequencing identified this bacterium as a member of the gram-negative genus *Aliiglaciecola. Aliiglaciecola* (meaning “the other inhabitant of ice”) is a cold-adapted genus with species from diverse marine locations, differentiated from its sister genus *Glaciecola* by virtue of physiological attributes such as fatty acid composition, thermotolerance, and nitrate reduction (6–8). The metabolic profile of this genus, and therefore, their role as algal symbionts is not yet fully understood (9).

The bacterium was isolated from *Ulva* biomass that was collected using aseptic techniques from the shoreline at Kulim Park in Tauranga, New Zealand (37.6652° S, 176.1488° E). As previously described (10) and made available in detail (see https://doi.org/10.6084/m9.figshare.28821062.v1), 10% (wt/vol) of *Ulva* biomass was used to inoculate marine minimal seawater media supplemented with trace elements (11). The culture was grown in a shaking incubator (200 rpm) at 25°C. Cultures were incubated for a period of 10 weeks. Growth from liquid culture was spread onto BD Difco marine agar 2216. Repeated single colony selection from marine agar was undertaken until a clonal strain was isolated, as determined by its uniform colony appearance and growth rates upon re-streaking. A stock of this strain was made by culturing the strain in BD Difco marine broth 2216 at 25°C, adding glycerol (25% [vol/vol]), and storing at −80°C. A single colony was used to inoculate 5 mL of Zobell marine broth, which was incubated for 72 h at 25°C with shaking. This culture was centrifuged to harvest cells for genomic DNA extraction, which was performed using the Wizard Genomic DNA Purification Kit (Promega, gram-negative bacteria protocol). Genome sequencing was completed by SeqCenter LLC (Pennsylvania, USA). Sample libraries were prepared using the Illumina DNA Prep kit and IDT 10 bp UDI indices and sequenced on an Illumina NextSeq 2000, producing 2 × 151 bp reads. Demultiplexing, quality control, and adapter trimming were performed with bcl-convert (v3.9.3) (12). Short-read assembly was performed with Unicycler (Version 0.4.8) (13). This produced an assembly containing 40 contigs with an *N_50_* value of 361,374 bp. Assembly statistics were recorded with QUAST (v5.0.2) (14). Contigs were annotated with the NCBI Prokaryotic Genome Annotation Pipeline (PGAP v6.5) (15). All programs were run under default parameters unless otherwise stated. Sequencing, assembly statistics, and genome features are summarized in Table 1.

**TABLE 1:** Assembly statistics and genome information for *Aliiglaciecola* sp. nov. SL4

Parameter	Data for *Aliiglaciecola* sp. nov. SL4
Sequencing and assembly	
No. of Illumina reads	866,049
Total no. of Illumina bases	323,864,299
Average coverage	28.69×
Genome description	
Genome size (bp)	4,375,838
G + C content	40.5%
Genes (total)	3,845
Coding sequences (total)	3,793
No. of proteins	3,759
No. of pseudogenes	34
No. of rRNAs	2
No. of tRNAs	46
No. of ncRNAs^*a*^	4

^*a*^ ncRNAs, non-coding RNAs.

The *Aliiglaciecola* genus was assigned on the basis of GTDB-tk v.2.3.2 (16) analysis, showing an average nucleotide identity of 82.52% with the bacterium *Aliiglaciecola lipolytica* strain D2R05, which was isolated from coastal seawater at Canoe Beach, Nahant, MA, USA (RefSeq number GCF_018860635.1). We designated this isolate as SL4 (for “sea lettuce 4”). The observed capacity for ulvan utilization, a major component of *Ulva* cell walls, indicates that *Aliiglaciecola* sp. nov. SL4 is likely an *Ulva*-associated bacterium; however, further investigation is required to validate this hypothesis.

## Data Availability

All data can be found in the NCBI BioProject accession number PRJNA1142097, including raw reads (SRA run number SRX26698477). This Whole Genome Shotgun project has been deposited at GenBank under the accession JBGOSE000000000. The version described in this paper is version JBGOSE010000000. Marine minimal medium components for seaweed-associated bacterial culture are publicly available through Figshare at https://doi.org/10.6084/m9.figshare.28821062.v1.
